# Outgroup, alignment and modelling improvements indicate that two TNFSF13-like genes existed in the vertebrate ancestor

**DOI:** 10.1007/s00251-016-0967-1

**Published:** 2017-01-09

**Authors:** Anthony K . Redmond, Rita Pettinello, Helen Dooley

**Affiliations:** 1 School of Biological Sciences, University of Aberdeen, Aberdeen, AB24 2TZ UK; 2 Centre for Genome-Enabled Biology & Medicine, University of Aberdeen, Aberdeen, AB24 2TZ UK; 3 Dept. Microbiology & Immunology, Institute of Marine & Environmental Technology, University of Maryland School of Medicine, 701 E. Pratt Street, Baltimore, MD21202 USA

**Keywords:** TNFSF13, BAFF, APRIL, BALM, Vertebrate evolution, Phylogenetics

## Abstract

The molecular machinery required for lymphocyte development and differentiation appears to have emerged concomitantly with distinct B- and T-like lymphocyte subsets in the ancestor of all vertebrates. The TNFSF superfamily (TNFSF) members BAFF (TNFSF13/Blys) and APRIL (TNFSF13) are key regulators of B cell development survival, and activation in mammals, but the temporal emergence of these molecules, and their precise relationship to the newly identified TNFSF gene BALM (BAFF and APRIL-like molecule), have not yet been elucidated. Here, to resolve the early evolutionary history of this family, we improved outgroup sampling and alignment quality, and applied better fitting substitution models compared to past studies. Our analyses reveal that BALM is a definitive TNFSF13 family member, which split from BAFF in the gnathostome (jawed vertebrate) ancestor. Most importantly, however, we show that both the APRIL and BAFF lineages existed in the ancestors of all extant vertebrates. This implies that APRIL has been lost, or is yet to be found, in cyclostomes (jawless vertebrates). Our results suggest that lineage-specific gene duplication and loss events have caused lymphocyte regulation, despite shared origins, to become secondarily distinct between gnathostomes and cyclostomes. Finally, the structure of lamprey BAFF-like, and its phylogenetic placement as sister to BAFF and BALM, but not the more slowly evolving APRIL, indicates that the primordial lymphocyte regulator was more APRIL-like than BAFF-like.

The TNF superfamily (TNFSF) cytokines BAFF (TNFSF13b/B cell activating factor/BLyS) and APRIL (TNFSF13/A proliferation-inducing ligand) are key regulators of B cell development, activation and survival in mammals (Mackay et al. 2003; Mackay and Leung 2006). Both BAFF and APRIL have been identified in teleost fishes (Glenney and Wiens 2007), along with a novel TNFSF member, BALM (BAFF and APRIL-like molecule), which shares similarity to both BAFF and APRIL. More recently, BAFF-like genes have been cloned from cartilaginous fishes (Ren et al. 2011; Li et al. 2012; Li et al. 2015) and lamprey (Das et al. 2016). APRIL has not yet been found in cartilaginous or jawless fishes, although the lamprey BAFF-like gene appears to share many APRIL-like characteristics (such as a positively charged, basic N-terminal end)(Das et al. 2016). The existence of a shared gene family governing lymphocyte regulation in the distinct adaptive immune systems of gnathostomes (based on antibodies, T cell receptors and major histocompatibility complex) and cyclostomes (based on variable lymphocyte receptors) adds significant support to the view that distinct B- and T-like lymphocyte lineages predate the emergence of these groups (Guo et al. 2009; Flajnik 2014; Das et al. 2016). Understanding the genetics and evolution of lymphocyte regulation in the jawed and jawless vertebrate adaptive immune systems is impossible at present, however, as the timing of emergence of the BAFF and APRIL lineages, as well as their precise kinship with BALM, are not yet fully clear. Here, to tackle this problem, we build upon the individual efforts of previous studies by incorporating improved sampling of important taxa and genes (see Zwickl and Hillis, 2002), testing the fit of alternative alignments and substitution models, as well as applying relaxed clock phylogenetic models (Drummond et al. 2006) to assess relationships between genes while avoiding inclusion of distant, and potentially biasing (Philippe et al. 2005; Pisani et al. 2015), outgroups.

As improved sampling of important taxa and genes can aid phylogenetic inference (Zwickl and Hillis 2002), we assembled a new dataset to best test the relationships between BAFF, APRIL and BALM. This was based on the study of Das et al. (2016), because this included TWEAK, BALM, lamprey BAFF-like and invertebrate TNFSF family members, as well as that of Li et al. (2015), as this included vertebrate EDA, the closest known outgroup to the TNFSF13 group (Glenney and Wiens 2007). We also searched for hagfish (*Eptatretus burgeri*) TNFSF13 family homologues in the Vertebrate TimeCapsule EST database (Takechi et al. 2011). This returned no obvious TNFSF13 homologues, but this dataset is relatively small compared to most modern RNA-seq studies and as such may be incomplete. We used three different alignment methods; PRANK, to correctly infer insertions and deletions (which can help to minimize alignment of non-homologous residues between sequences, and hence phylogenetic error) (Löytynoja and Goldman 2008), as well as MAFFT v6 (Katoh and Toh 2008) and ClustalW v2 (Larkin et al. 2007). Default settings in Mumsa v1.0 (Lassmann and Sonnhammer 2005) were used to rank alignments, revealing that the PRANK alignment was the best scoring (Table 1), and hence this was used for the main analysis. The CLUSTAL and MAFFT alignments were also analysed to observe the effects of alignment perturbation on the phylogenetic analysis. This was deemed to be of special significance here as our dataset and alignment are not identical to those used in previous studies (Glenney and Wiens 2007; Ren et al. 2011; Li et al. 2012; Li et al. 2015; Das et al. 2016). All alignments were manually curated to remove uninformative sites present in only one species. Modifications of the PRANK alignment were also used to test the effects of using only TWEAK as outgroup or only analysing the TNF domain on phylogenetic inference.

**Table 1:** Alignment and model selection statistics

Alignment	MUMSA rank	Best-fitting model
PRANK	1	JTT + Г
MAFFT	2	WAG + Г + F
CLUSTAL	3	WAG + Г + F
PRANK (no EDA)	−	JTT + Г + I
PRANK (TNF only)	−	LG + Г

Additionally, as poorly fitting substitution models, including those which do not account for rate variation across sites (Yang 1996), may generate branching artefacts, best-fit amino acid substitution models were selected for each alignment (Table 1) based on the Bayesian Information Criterion (BIC) in IQ-tree v1.4.4 (Nguyen et al. 2015). Bayesian phylogenetic analyses were performed in BEAST v1.8.3 (Drummond et al. 2012) using an uncorrelated lognormal relaxed molecular clock model (Drummond et al. 2006) to estimate the position of the root while accommodating rate variation between taxa (e.g. Macqueen et al. 2014; Zou et al. 2015), allowing the monophyly of BAFF, BALM and APRIL to be formally tested without the inclusion of many distant, and potentially biasing (Philippe et al. 2005; Pisani et al. 2015), outgroups. A Yule speciation prior (Yule 1925; Gernhard 2008) and the best-fit amino acid substitution model were also specified. Two Markov chain Monte Carlo runs were performed for each analysis, with burn-in removed and chains combined in LogCombiner v1.8.3 once convergence, mixing and effective sample sizes were sufficient (assessed using Tracer v1.6). Maximum clade credibility trees were generated in TreeAnnotator v1.8.3. This rigorous phylogenetic approach allowed us to establish the following:

## BALM is a definitive TNFSF13 family member

**Fig. 1:**
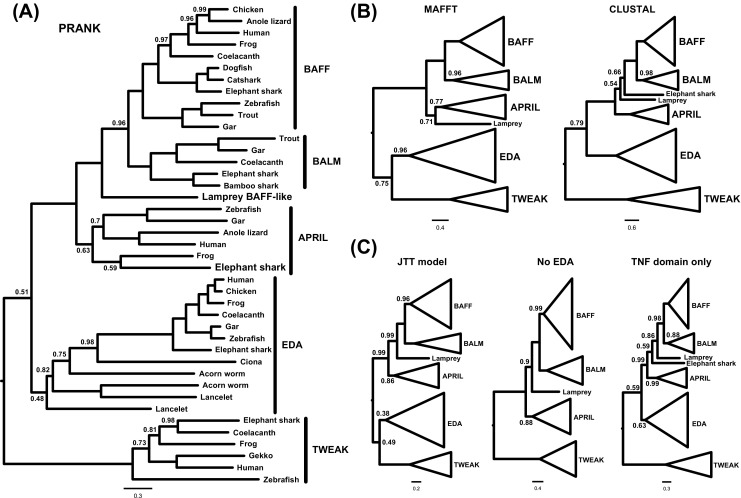
Phylogenetic analysis of the TNFSF13 family. **a** Full topology under the best-fitting model for both the PRANK alignment. **b** Collapsed phylogenies for the CLUSTAL and MAFFT alignments, under their best fitting models, show the impact of lower quality alignments. **c** Collapsed phylogeny for the PRANK analyses using either the poorly fitting JTT model, TWEAK alone as outgroup or only the TNF domain. In all cases, posterior probabilities are only reported where support is less than maximal. Accession numbers of sequences used in analyses: lamprey, *Petromyzon marinus* (BAFF/BALM-like: from Das et al. (2016)); elephant shark, *Callorhinchus milii* (APRIL?: AFP08081.1, BAFF: XP_007891666.1, BALM: AFP04129.1, EDA: XP_007893194.1, TWEAK: AFP92131.1); human, *Homo sapiens* (APRIL: O75888.1, BAFF: Q9Y275.1, EDA: Q92838.2, TWEAK: BAE16557.1); frog, *Xenopus laevis* (APRIL: NP_001267524.1, BAFF: AGN49363.1) and *Xenopus tropicalis* (EDA: XP_002934940.1, TWEAK: XP_012809319.1); chicken, *Gallus gallus* (BAFF: AAM90951.2, EDA: XP_003641179.2); anole lizard, *Anolis carolinensis* (APRIL: XP_008120421.1, BAFF: XP_003215395.2); bamboo shark, *Chiloscyllium plagiosum* (BALM: ADZ54859.1); catshark, *Scyliorhinus canicula* (BAFF: HG326662.1); dogfish, *Squalus acanthias* (BAFF: CCD04084.1); coelacanth, *Latimeria chalumnae* (BAFF: XP_005997065.1, BALM: XP_005997217.1, EDA: XP_005997183.1, TWEAK: XP_005999828.1); zebrafish, *Danio rerio* (APRIL: NP_001161936.1, BAFF: NP_001107062.1, EDA: NP_001108537.1, TWEAK: NP_001070075.1); trout, *Oncorhynchus mykiss* (BAFF: ABC84582.1, BALM: NP_001118038.1); gar, *Lepisosteus oculatus* (APRIL: XP_006627483.1, BAFF: XP_006639318.1, BALM: XP_006632891.1, EDA: XP_006632890.1); gecko, *Gekko japonicus* (TWEAK: XP_015277891.1); ciona, *Ciona intestinalis* (EDA-like: XP_002129711.1); acorn worm, *Saccoglossus kowalevskii* (EDA-like: XP_006826056.1 and XP_006821717.1); lancelet, *Branchiostoma floridae* (EDA-like: XP_002592907.1 and XP_002592910.1)

BALM has recently been shown to exist in a number of vertebrate lineages beyond bony fishes, but appears to be lost in tetrapods (Das et al. 2016). Its exact relationships to BAFF and APRIL, or other closely related TNFSF genes such as EDA (Glenney and Wiens 2007), have not yet been resolved however. Our relaxed clock rooting analyses consistently place EDA, or EDA and TWEAK (MAFFT alignment), as sister to BAFF, BALM and APRIL, revealing that BALM is a definitive TNFSF13 family member, which split from BAFF in the gnathostome ancestor (posterior probability ≥0.96; Fig. 1a, b). We propose the name TNFSF13c for the gene encoding BALM, in keeping with the established nomenclature in the TNFSF13 family.

## The APRIL lineage existed in the ancestor of vertebrates

The PRANK alignment indicates that the lamprey BAFF-like gene (Das et al. 2016) is co-orthologous to BAFF and BALM (PP = 1.00; Fig. 1a). In this analysis, gnathostome APRIL falls sister to this clade (PP = 1.00; Fig. 1a), revealing that APRIL has been lost, or is yet to be found, in cyclostomes. This means that two TNFSF13 genes existed in the vertebrate ancestor, as predicted by Collette et al. (2003), rather than a single gene as recently proposed by Das et al. (2016).

The MAFFT alignment places lamprey BAFF-like as sister to gnathostome APRIL (PP = 0.71), while the CLUSTAL alignment places it as sister to BAFF, BALM and elephant shark ‘BAFFb’ (Das et al. 2016) (PP = 0.66; Fig. 1b). Compared to the PRANK alignment, neither of these poorer-scoring alignments can place the lamprey BAFF-like sequence with high statistical support, and importantly, are not at odds with the above conclusion that at least two TNFSF13 genes existed in the ancestor of vertebrates.

Interestingly, the PRANK and MAFFT alignments place elephant shark ‘BAFFb’ (Das et al. 2016) in the gnathostome APRIL group, suggesting that this gene may be cartilaginous fish APRIL. Support for this hypothesis is weak (PP = 0.59–0.77; Fig. 1b), however, and, as mentioned above, is not supported by the CLUSTAL alignment, which suggests it may be a novel TNFSF13 family gene that is sister to gnathostome BAFF and BALM (PP = 0.66; Fig. 1b).

Of the most complete TNFSF13 family studies to date, our analyses are in general agreement with the results of Li et al. (2015) where applicable, but less so with those of Das et al. (2016). To explore the source of this discrepancy, we considered the differences between these studies and the analyses performed here. We have already accounted for variation in alignment methods used in the different studies, and from this it appears that a CLUSTAL alignment (Glenney and Wiens 2007; Ren et al. 2011; Li et al. 2012; Das et al. 2016), the worst performing method for our dataset (Table 1), may explain the weakly supported placement of lamprey BAFF-like, and the relatively unlikely placement of elephant shark ‘BAFFb’, in the study of Das et al. (2016), but not the poorly resolved relationship between BAFF and BALM, or the paraphyly of APRIL. We next analysed our PRANK alignment without permitting rate variation across sites, as was the case in most previous studies (Glenney and Wiens 2007; Ren et al. 2011; Li et al. 2012; Das et al. 2016), using the JTT model as applied by Das et al. (2016) but this impacted only statistical supports rather than branching orders (Fig. 1c), revealing the TNFSF13 phylogeny as relatively robust to model misspecification (see also Li et al. 2015). Despite vertebrate EDA being the most likely sister group to the TNFSF13 family (Glenney and Wiens 2007), this was not included in many previous datasets (Ren et al. 2011; Li et al. 2012; Das et al. 2016). Das et al. (2016) included TWEAK, however, another closely related TNFSF gene, and by excluding EDA we found that using TWEAK as the outgroup did not majorly impact the TNFSF13 phylogeny (Fig. 1c). Interestingly, it is not entirely clear from our analyses whether EDA alone or a clade containing both EDA and TWEAK is sister to the TNFSF13 family (Fig. 1). An alignment using only EDA as outgroup was not analysed here as our results are in keeping with those of Li et al. (2015) where this was previously performed. Finally, while most studies appear to have used full-length sequences (Glenney and Wiens 2007; Ren et al. 2011; Li et al. 2012; Li et al. 2015), Das et al. (2016) analysed only the TNF domain. This decision will have helped to avoid homoplasy in the rest of the dataset, but will also have reduced the total amount of data available for analysis. In our PRANK alignment, homoplastic misalignment should already be minimized, but we reanalysed this alignment over the TNF domain alone for the sake of comparison. This placed elephant shark ‘BAFFb’ as sister to the clade containing BAFF, BALM and lamprey BAFF-like with weak support (PP = 0.59; Fig. 1c), but otherwise had minimal effect on the resultant topology. While this placement of cartilaginous fish ‘BAFFb’, and its placement in the CLUSTAL analysis, is less parsimonious than its affinity for APRIL, Das et al. (2016) found it to be structurally most similar to BAFF. In light of this incongruence, we suggest this sequence is best treated as a rogue taxon at this point. While our search failed to pinpoint a single source for the discrepancy between previous studies, it may be that a combination of factors contributed to the paraphyly of APRIL and the poor resolution of the precise kinship between BAFF and BALM in the Das et al. (2016) study. This highlights the importance of jointly considering outgroup selection, alignment quality, rate variation across sites and well-fitting substitution models.

## The TNFSF13 family has ancient, APRIL-like origins

**Fig. 2:**
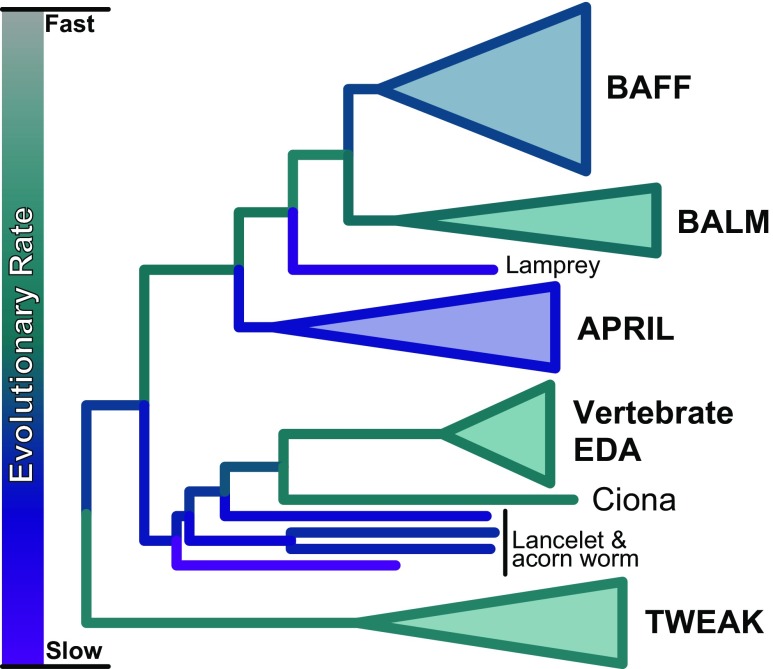
Phylogeny from Fig. 1a coloured by evolutionary rates inferred in the BEAST analysis

Of the jawed vertebrate TNFSF13 genes, APRIL is the slowest evolving on average (Fig. 2), suggesting that APRIL is less divergent from the ancestral TNFSF13 gene compared to BAFF and BALM. Further, Das et al. (2016) found that lamprey BAFF-like, which is even more slowly evolving than APRIL (Fig. 2), has structural similarities to APRIL with its positively charged, basic N-terminus. Taking the above together with its phylogenetic placement as sister to BAFF and BALM, it seems likely that at the very least the N-terminal end of the ancestral TNFSF13 family gene was more akin to APRIL than to BAFF or BALM.

**Fig. 3:**
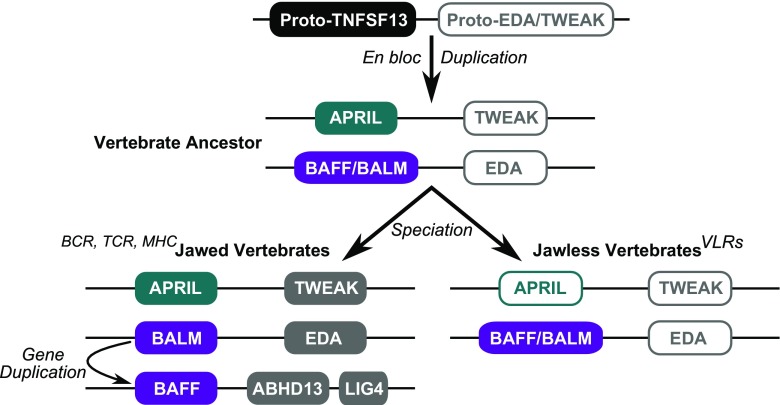
Simplified evolutionary scenario for the origin of the TNFSF13 repertoires in jawed and jawless vertebrates. *White filled boxes* indicate uncertainty of presence, relationships or timing of duplication; it is not yet clear whether APRIL has been lost in jawless vertebrates or has simply not been found yet. For jawed and jawless vertebrates, the genes and gene orders shown are proposed ancestral states

The synteny data of Das et al. (2016) are consistent with possible linkages between TWEAK and APRIL, and between EDA and BALM, in the gnathostome ancestor (Fig. 3). Based upon our phylogenetic analyses, which indicate close relationships between APRIL and BALM, and potentially TWEAK and EDA, it might be inferred that these loci are derived from an ‘en bloc’ duplication (Fig. 3). This may have been preceded by tandem duplication of an ancestral TWEAK/EDA/TNFSF13-like gene; however, other TNFSF genes, or gene blocks, may also be derived from this initial local duplication. Most parsimoniously, duplication of at least the BALM locus (in this case housing the BAFF/BALM ancestor gene) to a new location in the ancestor of jawed vertebrates would have produced BAFF (Fig. 3). We would therefore predict that the cyclostome BAFF-like gene will share synteny with BALM, not with BAFF.

Invertebrate sequences group with EDA in our phylogenetic analyses, albeit with low support (Fig. 1a). If this placement is correct then it indicates that at least one TNFSF13 gene has existed since the emergence of deuterostomes. The affinity of the invertebrate sequences to vertebrate EDA may be a branching artefact, however, as the branching order of invertebrate sequences suggests that at least three EDA lineages exist in invertebrates and require recurrent loss events (Fig. 1a), and both the tunicate branch (ciona EDA) and the branch leading to vertebrate EDA are quite long, each reaching far from their subtending node, and share highly similar evolutionary rates relative to the rest of the tree (Fig. 2), both of which are potential indicators of branching artefacts (e.g. Philippe et al. 2005).

## Lymphocyte regulation has become secondary distinct in jawed and jawless vertebrates

As our results suggest that lamprey BAFF-like is most likely co-orthologous to BAFF and BALM, it might reasonably be expected to be functionally equivalent to both, or either one, of these genes (Force et al. 1999). This calls for further studies of BALM and lamprey BAFF-like to determine their functional significance in lymphocyte regulation. Together with the probable loss of APRIL from lamprey and hagfish, there appears to be no extant one-to-one TNFSF13 family orthologs shared between jawed and jawless vertebrates, intimating that lineage-specific gene duplication and loss events have caused lymphocyte regulation to become secondarily distinct, at least on a genetic level, between these two major vertebrate lineages and adaptive immune strategies (Fig. 3).
